# Increasing trend in hypertension prevalence among Korean adolescents from 2007 to 2020

**DOI:** 10.1186/s12889-024-18093-w

**Published:** 2024-02-26

**Authors:** Peong Gang Park, Eujin Park, Hee Gyung Kang

**Affiliations:** 1 Departments of Pediatrics, Seoul National University Children’s Hospital, Seoul, Korea; 2 Departments of Pediatrics, Seoul National University College of Medicine, Seoul, Korea; 3 Departments of Pediatrics, Korea University Guro Hospital, Gurodong-ro, Guro-gu, Seoul, 08308 Korea

**Keywords:** Hypertension, Adolescent, Cross-sectional survey, Blood pressure

## Abstract

**Background:**

The purpose of this study was to examine the prevalence of hypertension in Korean adolescents, its long-term trends, and factors associated with the development of hypertension.

**Methods:**

Data of the Korea National Health and Nutrition Examination Survey (KNHANES) from 2007 to 2020 were combined into three time periods (2007–2011, 2012–2016, and 2017–2020). A total of 11,146 Korean adolescents aged 10–18 were included in the analysis. The definition of hypertension was based on the 2017 American Academy of Pediatrics guidelines for hypertension.

**Results:**

The age-adjusted prevalence of hypertension was 5.47%, 7.85%, and 9.92% in 2007–2011, 2012–2016, and 2017–2020, respectively. Long-term trend analysis using Joinpoint analysis over the observation period showed a significantly increasing trend in hypertension prevalence with a mean annual percentage change of 6.4%. Boys, those aged 13–15, those aged 16–18, overweight/obese, and those living in urban areas were more likely to develop hypertension (OR 1.980, 1.492, 3.180, 2.943, and 1.330, respectively).

**Conclusion:**

The prevalence of hypertension in Korean adolescents was higher than the global prevalence of hypertension and showed an increase over a 13–year period. Targeted strategies for prevention and early detection of hypertension are needed in this population.

## Introduction

Hypertension in children and adolescents is strongly associated with clinical and subclinical cardiovascular diseases in adulthood and end organ damage including the kidneys [1–3]. Individuals with persistent elevation of blood pressure from childhood have an increased risk of carotid atherosclerosis and an accelerated atherosclerotic process in adulthood [4, 5]. Hypertension in children and adolescents is associated with increased carotid intima-media thickness, which is known to increase the incidence of left ventricular hypertrophy [6, 7]. Eventually, these subclinical cardiovascular diseases from childhood increase the risk of cardiovascular events in adults with hypertension [8–10]. Several studies have also reported a link between pediatric hypertension and kidney damage indicated by microalbuminuria [11]. Therefore, clinicians are required to diagnose and manage childhood hypertension early to prevent related complications and improve their overall cardiovascular and kidney function [2, 12–14].

The prevalence of childhood hypertension is estimated to be 4% [15, 16]. However, it is important to note that the prevalence of hypertension in children may be higher in certain racial and ethnic groups due to genetic and socioeconomic factors. Additionally, regional and cultural preferences in diet can contribute to significant variations in the prevalence of hypertension among children and adolescents [16]. Data from the U.S. National Health and Nutrition Examination Survey (NHANES) show that the prevalence of hypertension among children aged 13–17 years is higher among non-Hispanic blacks than among non-Hispanic whites with a prevalence ratio of 2.03 [95% confidence interval (CI), 1.01–4.07] [17, 18]. In previous studies, the prevalence of hypertension in Africa, Europe, and the Western Pacific was higher than the global average [16, 19, 20]. However, data regarding the prevalence of pediatric hypertension in Asian populations are lacking [21]. Using data from the Korea National Health and Nutrition Examination Survey (KNHANES) from 2007 to 2020, we aimed to examine the prevalence and long-term trends of hypertension in Korean adolescents.

## Methods

### Study population and data collection

This study used the annual KNHANES data from 2007 to 2020 to estimate the prevalence of hypertension in adolescents aged 10–18 years. The KNHANES began in 1998 and has been an ongoing program of the Korea Disease Control and Prevention Agency (KCDC) for the past 20 years. It uses a multistage cluster probability sampling design to select a representative sample of the Korean civilian non-institutionalized population. Detailed information of this survey has been described elsewhere [22]. The KNHANES is weighted to account for the complex survey design and to produce nationally representative estimates based on the latest population and housing census. The survey included a health interview and physical examination; participants aged 10 years or older underwent blood pressure measurements. Blood pressure was measured on the right arm three times at 30-second intervals after a 5-minute rest period using uniform equipment and a cuff appropriate for the size of the arm; the mean of the second and third blood pressure measurements was used. Blood pressure was measured with a mercury (KNHANES 2007–2019) or non-mercury (KNHANES 2020) auscultatory sphygmomanometer, comparable to the mercury sphygmomanometer described in the 2018 Universal Protocol for Blood Pressure Measurement, with a maximum allowable error of less than 1 mmHg [23, 24]. The need for Institutional Review Board approval was waived because of the nature of the study.

### Definitions

We applied the 2017 American Academy of Pediatrics guidelines for hypertension, which define hypertension as a systolic or diastolic blood pressure of ≥ 95th percentile in the age-, sex-, and height-specific chart for children younger than 13 years old, and ≥ 130/80 mmHg for adolescents aged 13 years or older [2, 25]. We used reference values of blood pressure from the Fourth Report of the United States National High Blood Pressure Education Program since there were no reference data for Korean children using auscultatory sphygmomanometers [26, 27].

We calculated the age-adjusted prevalence of hypertension by applying age-specific rates to the 2007 Korean age distribution. We also stratified the prevalence of hypertension according to sex, age group (10–12, 13–15 and 16–18 years), body mass index (BMI) category (underweight/fit or overweight/obese), and rural/urban classification [17]. Overweight/obese was defined as a BMI of ≥ 85th percentile in the age- and sex-specific BMI chart based on the 2017 Korean national growth charts [28]. Rural/urban classification was as follows: rural—Gangwon, Chungbuk, Chungnam, Jeonbuk, Jeonnam, Gyeongbuk, Gyeongnam, and Jeju; urban—Seoul, Gyeonggi, Busan, Daegu, Incheon, Gwangju, Daejeon, Ulsan, and Sejong, as previously described [29].

### Statistical analyses

We used the US National Cancer Institute’s Joinpoint trend analysis software, a statistical method for analyzing trends using models with different segments connected at a “Joinpoint”, to test the significance of trends from 2007 to 2020 with the annual percentage change (APC) of hypertension prevalence and estimated *P* value [30, 31]. We also performed multivariable logistic regression using a survey-weighted generalized linear model with the logit link function, a statistical method for modeling binary outcomes with different weights for each observation, to identify the adjusted association of developing hypertension with the survey year, expressed as odds ratios (ORs) with 95% confidence intervals (CIs) [32]. Statistical analyses were performed using R (version 4.2.1; R Core Team, Vienna, Austria) with the *survey* package for weighted survey analysis. Statistical significance was defined at *P* < 0.05.

## Results

### Characteristics of the study participants

Table 1 presents the characteristics of the study participants from the KNHANES involving individuals aged 10–18 years (2007–2020; *n* = 11,146). Of the total participants, 53.5% (*n* = 5,941) were male, 21.7% (*n* = 2,421) were classified as overweight/obese, and 70.3% (*n* = 7,840) resided in urban areas. The data were divided into three time periods, 2007–2011 (*n* = 4,840), 2012–2016 (*n* = 3,773), and 2017–2020 (*n* = 2,533). There were no statistically significant differences in the sex distribution between the three time periods. However, statically significant differences were observed in the distribution of age, BMI categories, and region of residence. From 2007 to 2011 to 2017–2020, there was an increased in the proportion of individuals aged 16–18 years, those classified as overweight/obese, and those residing in urban areas, rising from 27.2 to 30.6%, 20.4 to 24.8%, and 68.4 to 73.1%, respectively (Table 1).

**Table 1:** Descriptive statistics of enrolled adolescents from Korea National Health and Nutrition Examination Survey in 2007–2011, 2012–2016, and 2017–2020^a^

Study year	2007–2011 (*n* = 4,840)	2012–2016 (*n* = 3,773)	2017–2020 (*n* = 2,533)	Overall (*n* = 11,146)	*P* value
Sex					0.747
- Boys	2,565 (53.0%)	2,010 (53.3%)	1,366 (53.9%)	5,941 (53.3%)	
- Girls	2,275 (47.0%)	1,763 (46.7%)	1,167 (46.1%)	5,205 (46.7%)	
Age group					0.002
− 10–12 years	1,872 (38.7%)	1,352 (35.8%)	954 (37.7%)	4,178 (37.5%)	
− 13–15 years	1,651 (34.1%)	1,306 (34.6%)	804 (31.7%)	3,761 (33.7%)	
− 16–18 years	1,317 (27.2%)	1,115 (29.6%)	775 (30.6%)	3,207 (28.8%)	
Body mass index category					< 0.001
- Underweight/Fit	3,852 (79.6%)	2,966 (78.6%)	1,904 (75.2%)	8,722 (78.3%)	
- Overweight/Obese	988 (20.4%)	807 (21.4%)	629 (24.8%)	2,424 (21.7%)	
Rural/urban classification					< 0.001
- Rural	1,529 (31.6%)	1,096 (29.0%)	681 (26.9%)	3,306 (29.7%)	
- Urban	3,311 (68.4%)	2,677 (71.0%)	1,852 (73.1%)	7,840 (70.3%)	

^a^Unweighted for all measures

### **Prevalence of hypertension in the 2007**–**2011, 2012**–**2016, and 2017**–**2020 study periods**

The age-adjusted prevalence of hypertension in 2007–2011, 2012–2016, and 2017–2020 was 5.47%, 7.85%, and 9.92%, respectively. In each time period, the prevalence of hypertension was higher among boys, individuals aged 16–18 years, those classified as overweight/obese, and those residing in urban areas compared with the overall prevalence of hypertension in the same period (Table 2). Moreover, from 2007 to 2011 to 2017–2020, the age-adjusted prevalence of hypertension continued to increase in both sexes, across all age and BMI categories, as well as among both rural and urban residents. Notably, the highest age-adjusted prevalence of hypertension in all three time periods was observed in the overweight/obese category, with rates of 10.43%, 16.62%, and 17.92% in 2007–2011, 2012–2016, and 2017–2020, respectively.

**Table 2:** Age-adjusted prevalence of hypertension by demographic and geographic characteristics from Korea National Health and Nutrition Examination Survey in 2007–2011, 2012–2016, and 2017–2020

Prevalence of hypertension	2007–2011 (*n* = 4,840)	2012–2016 (*n* = 3,773)	2017–2020 (*n* = 2,533)
Overall	5.47 (0.42)	7.85 (0.50)	9.92 (0.67)
Sex			
- Boys	7.51 (0.68)	9.40 (0.75)	12.73 (1.04)
- Girls	3.11 (0.45)	6.09 (0.65)	6.80 (0.81)
Age group			
− 10–12 years	2.32 (0.42)	4.66 (0.63)	7.24 (0.95)
− 13–15 years	4.57 (0.66)	7.16 (0.86)	7.47 (1.04)
− 16–18 years	9.90 (1.02)	12.35 (1.10)	15.19 (1.41)
Body mass index category			
- Underweight/Fit	4.19 (0.41)	5.45 (0.46)	7.39 (0.69)
- Overweight/Obese	10.43 (1.30)	16.62 (1.55)	17.92 (1.69)
Rural/urban classification			
- Rural	4.43 (0.71)	6.69 (0.89)	7.93 (1.27)
- Urban	5.92 (0.52)	8.36 (0.60)	10.75 (0.79)

### Long-term trends in the prevalence of hypertension

**Fig. 1:**
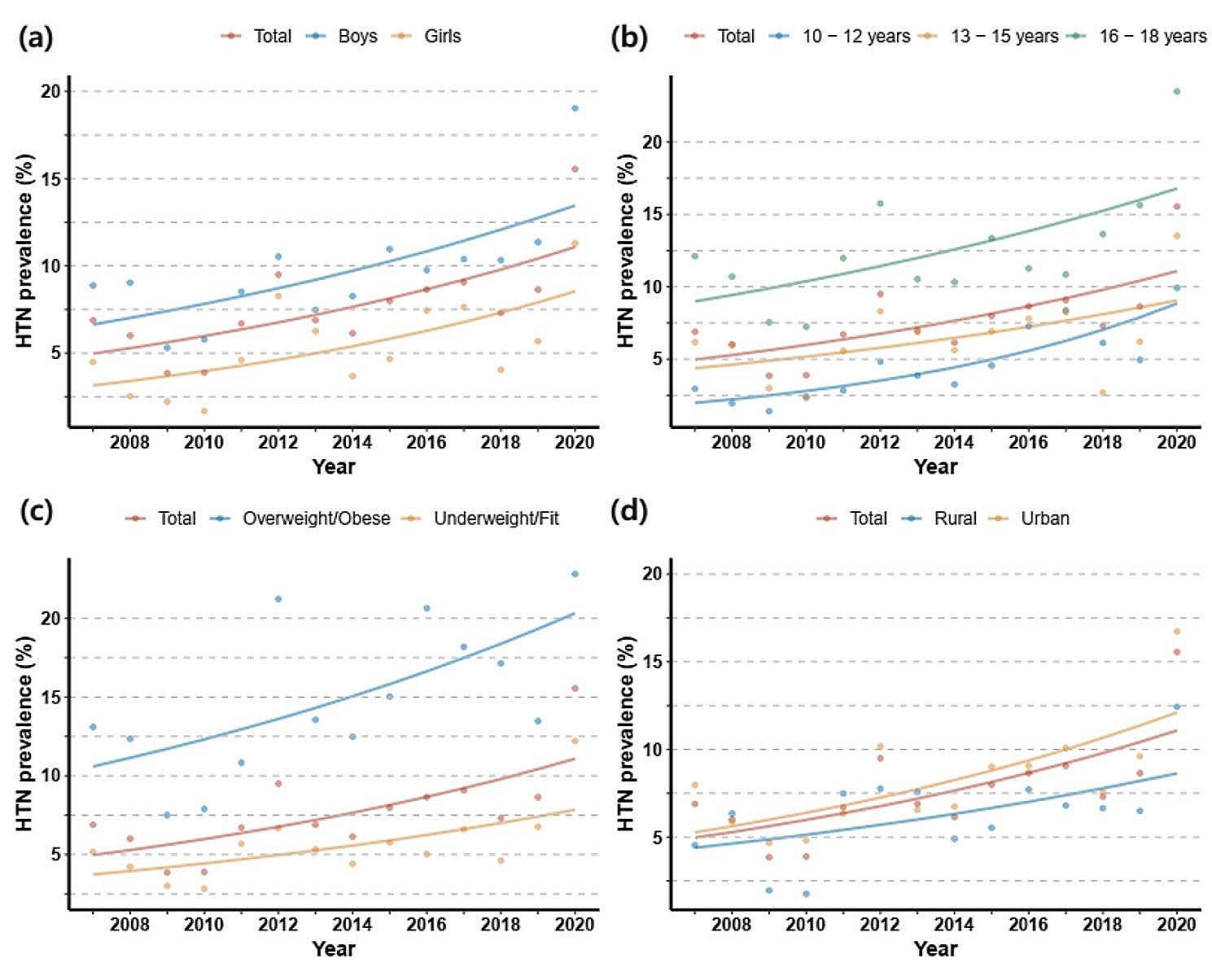
Trends of age-adjusted prevalence of hypertension among Korean adolescents. In total and by sex (**a**), age group (**b**), BMI category (**c**) and urban rural classification (**d**) (KNHANES, from 2007 to 2020). All prevalence measurement is age-adjusted using the 2007 Korean population, except for the age group subcategories

Long-term trend analysis using Joinpoint analysis over the observation period showed a significantly increasing trend in hypertension prevalence from 2007 to 2020, with a mean APC of 6.4%. This trend was also significant for both boys and girls (APC: 5.6% and 8.0%, respectively), for the age groups 10–12 years old and 16–18 years old (APC: 12.2% and 4.9%, respectively), adolescents with underweight/fit and obese/overweight BMI (APC: 5.9% and 5.1%, respectively), and for urban residents (APC: 6.6%), with *P* value < 0.05 for all trends (Table 3; Fig. 1).

**Table 3:** Annual percent change of hypertension prevalence by demographic and geographic characteristics from Korea National Health and Nutrition Examination Survey, trend for 2007 to 2020

	Annual percent change	*P*-Value
Total	6.4	0.003
Boys	5.6	0.003
Girls	8.0	0.019
10–12 years	12.2	0.000
13–15 years	5.8	0.056
16–18 years	4.9	0.010
Underweight/Fit	5.9	0.010
Overweight/Obese	5.1	0.012
Rural	5.3	0.075
Urban	6.6	0.002

All prevalence measurements were age-adjusted using 2007 Korean population data, except for age group subcategories

### Factors associated with the development of hypertension

A study-weighted logistic regression analysis showed that each increment in survey year was associated with significantly increased odds of developing hypertension, which was robust on adjusted analysis with consideration of covariates (age group, sex, BMI categories, and rural-urban classification; OR, 1.060; 95% CI, 1.053–1.067) (Table 4). Boys, individuals aged 13–15 years, those aged 16–18 years old, individuals classified as overweight/obese, and those residing in urban areas were found to have significantly higher odds of developing hypertension (OR 1.980, 1.492, 3.180, 2.943, and 1.330, respectively) (Table 4).

**Table 4:** Survey-weighted multivariable association of covariate with the development of hypertension

Risk Factor	aOR	95% CI
Year	1.060	1.053–1.067
Sex		
- Boys	1.980	1.873–2.092
- Girls	Ref	
Age group		
− 10–12 years	Ref.	
− 13–15 years	1.492	1.388–1.603
− 16–18 years	3.180	2.979–3.395
Body mass index category		
- Underweight/Fit	Ref.	
- Overweight/Obese	2.943	2.788–3.106
Rural/urban classification		
- Rural	Ref.	
- Urban	1.330	1.249–1.416

Abbreviations: aOR, adjusted odds ratio; CI, confidence interval. Multivariable analysis was conducted for all enrolled adolescents and controlled for age group, sex, BMI categories, rural-urban classification

## Discussion

The study found that the prevalence of hypertension in South Korean adolescents was higher than the global average and increased from 2007 to 2020. A recent systematic review reported that the global prevalence of hypertension in adolescents was 3.3% (95% CI, 2.69-3.97%) in 2000–2009 and 6.0% (95% CI, 4.38-7.91%) in 2010–2014 [16]. The study had similar proportions of boys and overweight/obese individuals, but unlike our study, it included children younger than 10 years, and differences in race and ethnicity may have contributed to the difference in prevalence [1, 33].

In this study, the age-adjusted prevalence of hypertension in the three time periods continued to be higher in boys than in girls: 7.51% vs. 3.11%, 9.40% vs. 6.09%, and 12.73% vs. 6.8% in 2007–2011, 2012–2016, and 2017–2020, respectively, and the overweight/obese individuals continued to be significantly higher than the underweight/fit individuals: 10.43% vs. 4.19%, 16.62% vs. 5.45%, and 17.92% vs. 7.39% in 2007–2011, 2012–2016, and 2017–2020, respectively. In addition, the age-adjusted prevalence of hypertension was higher among urban residents than rural residents: 5.92% vs. 4.43%, 8.36% vs. 6.69%, and 10.75% vs. 7.93% in 2007–2011, 2012–2016, and 2017–2020, respectively. Subsequent multivariable analysis reaffirmed that boys, children aged 13 years and older, overweight/obese individuals, and those living in urban areas were more likely to develop hypertension. These are well-established risk factors for pediatric hypertension, and these findings align with previous research [1, 34, 35]. Differences in the prevalence of hypertension with sex and age can be explained by changes in gonadal hormone levels and adiposity [36, 37]. The mechanisms of obesity-hypertension result from a combination of impaired sodium handling, sympathetic nervous system overactivation, oxidative stress, hemodynamic changes, and renal/endocrine dysfunction [38, 39]. These findings are consistent across studies conducted in Asian and Western countries. In a report on Chinese children and adolescents, boys, those aged 13 years and older, overweight/obese, and urban individuals were more likely to develop hypertension [19]. Similarly, in a study of U.S. adolescents using NHANES data, hypertension was more common in boys, those aged 15 years and older, and those who were overweight/obese [17].

Our data also confirms the upward secular trend in hypertension prevalence (from 2007 to 2020, with a mean APC of 6.4%). These findings are consistent with a recent systematic review that identified a positive secular trend in the global prevalence of pediatric hypertension over the past two decades [16]. However, these results have been inconsistent in several other studies. A recently reported study examining secular trends in the prevalence of hypertension in Japanese adolescents aged 12 to 18 years from 2000 to 2019 found that the prevalence of hypertension in boys was highest in 2005–2009, with a steady decline thereafter, while the prevalence of hypertension in girls did not change significantly during the study period. The authors noted that their findings may differ from the global prevalence of pediatric hypertension because of racial differences and the influence of lifestyle and living environment, including social and economic factors [40, 41]. When comparing their study with ours, the study had a lower proportion of girls, did not include adolescents aged 10–12 years but had a higher proportion of senior high school students, and had a lower proportion of overweight/obese individuals than our study, which may have contributed to the discrepancy in results between the studies. A meta-analysis of 13 articles published between 2005 and 2016 also reported that the prevalence of hypertension in Nigerian children and adolescents was 5.1% (95% CI, 2.9–8.6%), with a significant negative trend (Z = -0.89; α < 0.01) over the past 20 years [42]. However, this study did not provide detailed information on the characteristics of the study participants, making comparison with the present study difficult.

In an earlier study using KNHANES data, secular trends of increased prevalence of elevated blood pressure and hypertension in Korean adolescents were reported between the periods of 2007–2009 and 2013–2015 [43]. This trend has also been reported in Korean adults, where the prevalence and number of patients with hypertension steadily increased between 1998 and 2018 [44]. Additionally, familial aggregation of hypertension has been identified in Korean families, where children whose parents have hypertension are more likely to develop hypertension during childhood [45]. The association between a parental history of hypertension and blood pressure levels in adult offspring has been well documented in previous studies, with a meta-analysis reinforcing the concept that blood pressure tracks from childhood to adulthood and that an elevated blood pressure in childhood may help predict adult hypertension [46, 47]. Because of the adverse health consequences, there is a need to raise awareness and provide early intervention for adolescents with hypertension at the family level through community health education, blood pressure screening programs, good nutrition, and regular physical activity [2, 25].

This study has the strength of comprehensively showing the prevalence of hypertension among adolescents in Korea over a long period; however, several limitations should also be acknowledged. First, the definition of hypertension was based on three blood pressure measurements taken at a single visit. Second, data on treatment with antihypertensive agents were unavailable. Third, although validated according to the Universal Protocol for Blood Pressure Measurement in 2018, there were differences between mercury and non-mercury auscultatory sphygmomanometers between the devices used for measurements in 2007 through 2019 and those used in 2020. Fourth, we did not investigate the risk of hypertension based on perinatal factors, diet and life style changes, such as those that may have occurred during the coronavirus disease 2019 pandemic. These factors were not considered in the multivariable analysis, limiting our ability to establish a causal relationship between these factors and hypertension.

## Conclusion

Our data provides valuable insights into the prevalence of hypertension in a representative sample of Korean adolescents aged 10–18 years spanning from 2007 to 2020. We found that the prevalence of hypertension in this adolescent population was higher than the previously reported global prevalence of hypertension. Furthermore, our study revealed an upward trend in hypertension prevalence with higher rates observed among boys, adolescents aged 13 years and older, overweight/obese individuals, and urban residents. Considering the unfavorable health outcomes of childhood hypertension, our findings underscore the need for effective screening programs for at-risk populations.

## Data Availability

The datasets generated and analyzed during the current study are publicly available in the Korean National Health and Nutrition Examination Survey (http://knhanes.kdca.go.kr/).

## Abbreviations

NHANES: U.S. National Health and Nutrition Examination Survey
KNHANES: Korean National Health and Nutrition Examination Survey
KCDA: Korea Centers for Disease Control and Prevention Agency
BMI: Body mass index
APC: Annual percentage change
ORs: Odds ratios
CIs: Confidence intervals
